# The relationship between self-reported alcohol intake and the morbidities managed by GPs in Australia

**DOI:** 10.1186/1471-2296-7-17

**Published:** 2006-03-14

**Authors:** Elizabeth M Proude, Helena Britt, Lisa Valenti, Katherine M Conigrave

**Affiliations:** 1 School of Public Health, The University of Sydney, Sydney, New South Wales, Australia; 2 Drug Health Services, Royal Prince Alfred Hospital, Camperdown, New South Wales, Australia; 3 The University of Sydney General Practice Statistics and Classification Centre, Westmead Hospital, Westmead, New South Wales, Australia; 4 Disciplines of Medicine, The University of Sydney, Sydney, New South Wales, Australia; 5 Psychological Medicine, The University of Sydney, Sydney, New South Wales, Australia

## Abstract

**Background:**

One in five Australians consume alcohol at risky or harmful levels. Most (85%) attend a general practitioner at least once a year, giving opportunity for detecting and providing brief interventions for reducing alcohol-related harm. Historically, detection rates of problem drinking have been low in general practice, producing lower prevalence estimates of heavy drinking than expected from population surveys.

**Method:**

The BEACH program collects data from 100 consecutive patient consultations with 1000 GPs annually. For 40 consecutive encounters, GPs ask adult patients three questions on alcohol consumption (AUDIT-C). This paper reports the problems managed and treatments provided at encounters with heavy and non-heavy drinkers, grouped by their response to the 3rd question, and compares the two groups before and after standardisation for age and sex. Heavy drinking was defined as having 6 or more standard drinks at least once a week or more often.

**Results:**

Heavy drinking was reported by 7.3% patients overall; more prevalent among men (13.8%) than women (3.9%); and among Indigenous patients (18.5%). Prevalence was highest in young adults (18–24 years)(12.7%) and decreased with age. Patients from a non-English speaking background were less likely to be heavy drinkers. Heavy drinkers had more problems managed at encounters, more chronic problems, physical injuries and psychological problems (particularly depression) managed than non-heavy drinkers. They were less likely to have respiratory complaints, ischaemic heart disease or diabetes managed.

**Conclusion:**

Heavy drinkers are more likely than non- or light drinkers to see their GP for management of chronic problems, psychological problems and physical injuries. However, the wide range of morbidity managed in heavy drinkers means that relying on clinical impression alone to detect this group will not suffice and should be augmented with routine screening. Given the pressures of general practice, finding efficient methods of screening for alcohol problems remains a priority.

## Background

Fifteen per cent of the Australian population consume alcohol at risky levels, while another 5% are at high risk of damage from alcohol or are alcohol-dependent [1,2]. Alcohol misuse takes a toll on individual health. It also contributes to health care costs through general practice (GP) and emergency department presentations and hospital admissions. It can impact on the individual and family quality of life and can lead to early death.

Early and brief intervention has been demonstrated to be effective for management of excessive alcohol use and is cost-effective [3-5]. However, early intervention relies on active and accurate recognition of alcohol problems. In contrast as few as 30% of alcohol problems are said to be recognised by either GPs [6,7] or hospital doctors [8-10].

In Australia, GPs are the gatekeepers to the health care system. There are over 17,000 recognised GPs and about 1,500 registrars enrolled in general practice training programs. GPs are the most frequently consulted health professionals (85% attend a GP at least once in a given year) [11]. There are no patient lists, people being free to visit multiple GPs and multiple practices at any time. Remuneration is on a fee-for-service basis, but the Australian Government currently pays for 85–100% of a fee set by Government (the Medicare Schedule fee) for each service [12].

The availability of data from a continuous national study of general practice clinical activity provided us with the opportunity to investigate the prevalence of risky alcohol consumption among patients attending general practice, and investigate the relationship between self-reported alcohol intake and the morbidities managed by GPs in Australia.

## Method

The 'Bettering the Evaluation and Care of Health' (BEACH) program is a continuous national study of general practice activity in Australia; sampling and method have been described in detail elsewhere [11,13]. In summary, each year a random sample of practising GPs, drawn from the Australian Government records of GP services claims for payment through the universal health insurance scheme (Medicare), are invited to participate. Approximately 1,000 GPs participate annually, complete a questionnaire about themselves and their practice, and record patient morbidity and management details for each of 100 consecutive encounters on structured paper forms. Each participant's 100 encounters are then weighted according to the number of claims made through Medicare in the previous quarter.

The characteristics of the final GP sample are compared with those of the total sample frame, and post-stratification weighting applied for any under-representation of particular groups of GPs. Comparison of the age-sex distribution of patients in the final encounter sample with the age-sex distribution of patient at all encounters claimed through Medicare repeatedly demonstrates excellent precision [11].

We undertook a secondary analysis of a sub-sample from BEACH April 2001–September 2003. For 40 consecutive encounters out of their 100, each GP was instructed to ask the patient (if aged 18 years or over) questions regarding their usual alcohol consumption, using the first three items (AUDIT-C) from the WHO Alcohol Use Disorders Identification Test (AUDIT) [14]. The subsamples were placed successively per GP as either the first 40, the middle 40 or the last 40 in the pack of 100 forms [11,13]. The questions asked by the GP and the response options available are shown in Table 1. The GPs were provided with a standard drinks chart [15] to show to the patient when asking these questions.

**Table 1:** Questions asked of patients by the GPs.

**Question**	**Response categories**
How often do you have a drink containing alcohol?	Never Monthly or less Once a week/fortnight 2–3 times a week >4 times a week
How many standard drinks do you have on a typical day when you are drinking?	Number....
How often do you have 6 or more standard drinks on one occasion?	Never Less than monthly Monthly Weekly Daily or almost daily

This paper focuses on the third question, which was used to divide patients into two groups:

• Heavy drinkers: Patients who have six or more standard drinks (each of 10 g ethanol) on one occasion once a week or more often.

• Non-heavy drinkers: All other patients.

This allows detection of patients who regularly place themselves at acute risk of harm, and those who are daily heavy drinkers. By comparison, the Australian National Health & Medical Research Council (NHMRC) defines acute risk of harm as consumption of >7 drinks for males, or >5 drinks for females on any one occasion [16]. This screening question has been found to have high specificity (from 79% to 96%) for detecting drinking problems, and sensitivity ranging from 45% to 83% [17-20].

We classified patient reasons for encounter (RFEs) (up to three), problems managed (up to four), clinical treatments (up to two per problem) and therapeutic procedures (up to two per problem) according to the International Classification of Primary Care (ICPC-2) [21]. Chronic problems were defined according to O'Halloran *et al *[22]. Demographic details of the two groups were compared. Non-English speaking background (NESB) and Indigenous status of patient were self-reported. Analyses were performed using both the unadjusted and age-sex standardised data sets (standardised to the age-sex distribution of all GP encounters claimed through the Australian Government during 2001). Significance of differences in heavy drinking prevalence between selected sub-groups of patients (e.g. males and females) (Table 2) was tested with the chi squared test statistic (α = 0.01) with adjustment for the cluster sample design using STATA 8 [23]. Differences between the heavy and non-heavy drinkers are described in terms of rates of events per 100 encounters, or per 100 problems (Tables 3 and 4). Non-overlapping 95% confidence intervals, adjusted for the cluster study design, using SAS [24] indicate a significant difference between the groups.

**Table 2:** Prevalence of heavy drinkers by patient characteristic

**Patient variable**	Number (n)	Unadjusted descriptive		Age-sex standardised	
			
		Heavy drinkers (6+ at least weekly), variable specific rate (N = 5,753)		Heavy drinkers (6+ at least weekly), variable specific rate (N = 5,756)	
		
		Per cent of n	p-value*	Per cent of n	p-value*
**Sex**					
Males	4,238	13.8	p < 0.01		
Female	1,515	3.1	---		
**Age**					
18–24 years	878	12.7	p < 0.01		
25–44 years	2,250	9.5	---		
45–64 years	1,941	7.8	---		
65–74 years	476	4.3	---		
75+ years	208	1.7	---		
**Holds Health Care card**					
Yes	1,955	5.8	p < 0.01	7.7	p = 0.07
No	3,212	8.4	---	7.0	---
**Holds Veterans' Affairs card**					
Yes	164	5.2	p < 0.01	8.7	p = 0.18
No	4,900	7.3	---	7.2	---
**Non-English speaking background^(a)^**					
Yes	174	2.8	p < 0.01	3.3	p < 0.01
No	5,137	7.6	---	7.6	---
**Indigenous**					
Yes	173	18.5	p < 0.01	18.9	p < 0.01
No	5,580	7.2	---	7.2	---
**New to practice**					
Yes	733	10.5	p < 0.01	8.5	p = 0.02
No	4,901	7.0	---	7.1	---
**Daily smoker**					
Yes	2,406	17.5	---	16.1	---
No	3314	5.1*	---	5.3*	---

* P value for design based F statistic, which adjusts for the cluster sample design. Significant at α = 0.01.

(a) Defined as speaks another language as their first language at home

Note: CI – confidence interval; New to practice – not previously seen by a medical practitioner in this practice.

**Table 3:** The content of the encounters

	**Unadjusted descriptive**					**Age-sex standardised**				
	
**Variable**	**Heavy drinkers**			**Non-heavy drinkers**		**Heavy drinkers**			**Non-heavy drinkers**	
						
	**Rate per 100 encounters (n = 5,753)**	**95% CI**		**Rate per 100 encounters (n = 73,221)**	**95% CI**	**Rate per 100 encounters^(a) ^(*n *= 5,756)**	**95% CI**		**Rate per 100 encounters (n = 73,152)**	**95% CI**
	
**Reasons for encounter**	150.0	147.7–152.2	↓	155.7	154.5–156.9	156.60	152.7–160.4	--	155.0	153.8–156.2
**Problems managed**	151.1	148.6–153.6	↓	155.5	154.1–156.9	160.6	156.3–164.9	↑	154.1	152.7–155.5
New problem	58.8	56.7–60.9	↑	53.9	53.1–54.8	56.4	53.2–59.6	--	54.2	53.3–55.1
Work related	2.6	2.1–3.1	↑	1.3	1.1–1.5	1.6	1.2–2.0	--	1.4	1.2–1.6
Chronic problems	52.0	49.7–54.3	↓	56.6	55.4–57.7	62.0	57.3–66.6	↑	55.3	54.2–56.5
	
**Treatment type**	**Rate per 100 problems (n = 8,694)**	**95% CI**		**Rate per 100 problems (n = 113,885)**	**95% CI**	**Rate per 100 problems (n = 9,245)**	**95% CI**		**Rate per 100 problems (n = 112,735)**	**95% CI**
	
**Medications**	67.5	65.4–69.5	--	68.0	67.1–69.0	70.7	66.8–74.5	--	68.1	67.1–68.0
Prescribed	55.9	53.8–57.9	--	57.4	56.3–58.4	58.1	55.5–62.8	--	57.2	56.2–58.2
Advised OTC	5.4	4.8–6.0	--	5.2	5.0–5.5	4.7	3.9–5.6	--	5.4	5.1–5.6
GP-supplied	6.2	5.2–7.2	--	5.4	4.9–5.9	6.8	4.8–8.8	--	5.5	5.0–6.0
**Clinical treatment**	33.5	31.9–35.2	↑	26.8	25.9–27.6	32.1	30.0–34.2	↑	27.2	26.3–28.1
Counselling – alcohol*	5.7	5.0–6.4	↑	0.2	0.2–0.3	5.8	4.7–6.9	↑	0.2	0.2–0.3
Counselling – psychological*	5.0	4.3–5.6	↑	4.0	3.7–4.3	5.6	4.5–6.6	↑	4.0	3.7–4.3
Counsell/advice – smoking*	2.3	1.9–2.7	↑	0.9	0.8–1.0	2.2	1.6–2.7	↑	0.9	0.8–1.0
**Procedural treatment**	11.4	10.5–12.3	--	11.0	10.6–11.4	10.4	9.1–11.8	--	10.9	10.5–11.3
**Referrals**	8.6	7.9–9.2	--	8.0	7.7–8.2	7.7	6.7–8.7	--	8.0	7.8–8.2
Specialist	5.3	4.8–5.8	--	5.4	5.2–5.6	5.0	4.2–5.9	--	5.4	5.3–5.6
Allied health services	2.4	2.1–2.7	--	2.0	1.8–2.1	1.9	1.4–2.4	--	2.0	1.9–2.1
Emergency department	0.1	0.0–0.2	--	0.1	0.1–0.1	0.1	0.0–0.1	--	0.1	0.1–0.1
Hospital	0.4	0.3–0.6	--	0.3	0.3–0.3	0.5	0.3–0.7	--	0.3	0.3–0.3
**Pathology**	26.0	24.0–28.0	--	25.7	24.9–26.4	27.7	24.5–30.9	--	25.8	25.0–26.5
**Imaging**	6.6	5.9–7.3	--	6.0	5.8–6.2	6.2	4.9–7.4	--	6.1	5.9–6.3

Note: CI – confidence interval; work-related – in the opinion of the GP the problem is likely to be work related; new problem – first consultation by this patient for this problem with any medical practitioner.

↑ Significantly higher at encounters with heavy drinkers than at those with non-heavy drinkers

↓ Significantly lower at encounters with heavy drinkers than at those with non-heavy drinkers

-- No significant difference between the groups

**Table 4:** Problems managed – significant differences between groups in rates of management of problems in terms of ICPC-2 chapters & common problems managed.

**Common chapters and individual problems**	**Unadjusted descriptive**					**Age-sex standardised**				
		
	**Heavy drinkers (6+ at least weekly)**			**Non-heavy drinkers**		**Heavy drinkers (6+ at least weekly)**			**Non-heavy drinkers**	
						
	**Rate per 100 encounters (n = 5,753)^(a)^**	**95% CI**		**Rate per 100 encounters (n = 73,221)^(a)^**	**95% CI**	**Rate per 100 encounters (n = 5,756)^(a)^**	**95% CI**		**Rate per 100 encounters (n = 73,152)^(a)^**	**95% CI**
	
**Psychological**	**20.3**	**18.8–21.9**	↑	**12.7**	**12.3–13.2**	**22.1**	**19.8–24.5**	↑	**12.8**	**12.3–13.3**
Depression*	5.6	5.0–6.2	↑	4.5	4.3–4.7	6.7	5.5–7.9	↑	4.5	4.3–4.7
Chronic alcohol abuse	3.2	2.6–3.8	↑	0.1	0.1–0.1	2.9	2.2–3.5	↑	0.1	0.1–0.1
Acute alcohol abuse	1.7	1.4–1.7	↑	<0.1	0.0–0.1	1.8	1.2–2.3	↑	<0.1	0.0–0.1
Drug abuse	1.2	0.9–1.6	↑	0.5	0.4–0.6	0.9	0.6–1.2	--	0.6	0.4–0.7
Tobacco abuse	1.0	0.8–1.5	↑	0.4	0.4–0.5	1.1	0.7–1.5	↑	0.4	0.4–0.5
**Musculoskeletal**	**20.7**	**19.4–22.0**	--	**19.4**	**18.9–19.9**	**21.0**	**18.4–23.7**	--	**19.4**	**18.9–19.9**
Back complaint*	4.2	3.6–4.7	↑	3.1	2.9–3.3	3.1	2.5–3.7	--	3.2	3.0–3.4
Osteoarthritis*	2.0	1.7–2.4	↓	3.1	3.0–3.3	3.8	2.5–5.1	--	3.0	2.8–3.1
Fracture*	1.9	1.5–2.3	↑	0.9	0.8–0.9	1.8	1.0–2.7	--	0.9	0.8–1.0
Sprain/strain*	2.7	2.3–3.2	↑	1.6	1.5–1.8	1.8	1.4–2.1	--	1.7	1.6–1.9
**Circulatory**	**14.0**	**13.0–15.0**	↓	**19.7**	**19.1–20.2**	**17.7**	**15.3–20.0**	--	**18.9**	**18.3–19.4**
Hypertension*	8.5	7.7–9.3	↓	10.9	10.5–11.2	11.3	9.4–13.1	--	10.5	10.1–10.8
Ischaemic heart disease*	1.0	0.7–1.3	--	1.5	1.3–1.6	0.9	0.6–1.2	↓	1.4	1.3–1.5
**Skin**	**20.2**	**18.8–21.5**	↑	**16.1**	**15.6–16.6**	**16.9**	**15.1–18.7**	--	**16.1**	**15.6–16.6**
Laceration/cut	1.2	0.9–1.5	↑	0.6	0.5–0.7	0.7	0.5–0.9	--	0.6	0.6–0.7
Other skin symtpom/complaint	1.1	0.8–1.4	↑	0.5	0.5–0.6	1.0	0.4–1.6	--	0.6	0.5–0.6
**Respiratory**	**16.1**	**15.0–17.2**	↓	**18.6**	**18.1–19.2**	**15.9**	**14.2–17.7**	↓	**18.8**	**18.2–19.3**
**Endocrine & metabolic**	**10.9**	**9.9–11.8**	↓	**12.6**	**12.2–13.0**	**12.2**	**10.5–13.9**	--	**12.5**	**12.1–12.9**
Diabetes*	2.1	1.7–2.5	↓	3.6	3.5–3.8	2.6	1.7–3.4	↓	3.6	3.4–3.8
Gout	1.5	1.2–12.8	↑	0.5	0.5–0.6	1.3	0.8–1.8	↑	0.5	0.5–0.6
**Female Genital system**	**4.2**	**3.6–4.8**	↓	**8.4**	**8.0–8.8**	**8.0**	**6.7–9.2**	--	**8.8**	**8.4–9.2**
**Neurological**	**3.4**	**2.7–4.1**	↓	**4.3**	**4.2–4.5**	**4.0**	**2.9–5.0**	--	**4.3**	**4.2–4.5**
**Pregnancy and family planning**	**3.2**	**2.5–3.9**	↓	**5.1**	**4.8–5.3**	**4.6**	**3.6–5.5**	--	**5.2**	**4.9–5.5**
**Urological**	**1.9**	**1.5–2.2**	↓	**3.2**	**3.0–3.3**	**2.8**	**1.8–3.9**	--	**3.3**	**3.2–3.4**
**Digestive**	**11.5**	**10.6–12.3**	↑	**10.1**	**9.7–10.3**	**11.7**	**10.2–13.3**	--	**10.0**	**9.7–10.3**

(a) Only the ICPC-2 chapters and/or the individual problems within chapters for which significant differences were identified between the two groups are reported here.

↑ Significantly higher at encounters with heavy drinkers than at those with non-heavy drinkers

↓ Significantly lower at encounters with heavy drinkers than at those with non-heavy drinkers

-- No significant difference between the groups

* Includes multiple ICPC-2 code

### Statement of ethics clearance

The BEACH program was approved by the Health Ethics Committee of the Australian Institute of Health and Welfare and the Human Ethics Committee of the University of Sydney.

## Results

Over the 2.5 years, responses were received for 78,974 adult patients from 2,470 GPs.

### Demographics

Mean patient age was 52 years (range 18–102); and 38.8% were male. The prevalence of heavy drinking (6 drinks on one occasion at least weekly) was 7.3% (95% CI: 7.0–7.6), and was higher among men (13.8%) than in women (3.1%). Heavy drinking was most prevalent among young adults aged 18–24 years (12.7%), and decreased steadily with patient age group to only 1.7% among patients aged 75 years and over.

Both before and after age-sex standardisation, the prevalence of heavy drinking was significantly higher among patients who were from an English speaking background and among Indigenous patients. Eighty percent of the heavy drinkers identified by the screening question were more moderate or non-drinkers for most of the week with just episodic (e.g. weekend) heavier drinking. Only 20% of the heavy drinkers consumed 6 or more drinks daily or almost daily.

### Content of encounters

Using unadjusted data, patients classified as heavy drinkers presented to the GP with significantly fewer RFEs; had fewer problems managed at the encounter and fewer chronic problems, but significantly more new and work-related problems. After age-sex standardisation, only the number of problems and the number of chronic problems managed at encounters were significantly higher than at encounters with non-heavy drinkers (Table 3).

Both before and after age-sex standardisation there were no differences between the groups in terms of the rate per 100 problems managed in: medications prescribed, supplied or advised for over-the counter purchase; procedural treatments undertaken; referrals, pathology and imaging (in total and to specific service groups). However, both before and after adjustment, heavy drinkers received clinical treatments significantly more often relative to the number of problems managed at encounter. This difference was largely explained by higher rates of counselling about alcohol, about smoking and of provision of general psychological counselling at encounters with heavy drinkers (Table 3).

The differences between the groups in terms of their RFEs (results not reported) were closely reflected in the problems managed, so this paper focuses on the latter. Table 4 shows that heavy drinkers had significantly higher management rates of psychological problems overall, and more specifically of depression, chronic and acute alcohol abuse, drug abuse and tobacco abuse. All of these except drug abuse remained significant after age-sex standardisation. Apparent differences in rates of management of back complaints, fractures, and sprain/strains disappeared after adjustment.

A separate analysis, investigating the management rate of all physical injuries, demonstrated higher management rates in heavy drinkers (unadjusted 11.4 per 100 encounters 95% CI: 10.5–12.3 compared with 6.4 per 100, 95% CI: 6.1–6.6). These differences remained after adjustment (8.4 per 100 encounters, 95% CI: 7.2–9.7 compared with 6.6 per 100, 95% CI: 6.4–6.9) (results not tabled). Problems related to the skin (laceration/cut and other skin complaints in particular) were more frequently managed in heavy drinkers but no difference remained after adjustment.

Problems less often managed at encounters with heavy drinkers were those related to the respiratory, circulatory, female genital or reproductive system, and neurological, urological, and endocrine/metabolic systems. However after age-sex adjustment, the only difference that remained was the lower rate of respiratory problems. While circulatory problems as a whole no longer differed after adjustment, ischaemic heart disease was managed less often in heavy drinkers. Diabetes was less often managed and gout more often managed in heavy drinkers even after adjustment.

## Discussion

This study has demonstrated that heavy drinkers seen in general practice are commonly managed for injuries and for psychological problems, particularly depression. They also have more problems managed at their GP encounters. Even after age-sex standardisation, this effect remained. Further, 80% of the heavy drinkers identified by the screening question were episodic rather than regular heavy drinkers.

As expected, males were more likely than females and smokers more likely than non-smokers to be heavy drinkers. However, contrary to traditional stereotypes of problem drinkers, prevalence was higher among young adult patients than among middle-aged and older people. Further, after age-sex standardisation prevalence of heavy drinking was not higher among those who held a concessional health care card (a broad indicator of social disadvantage) than among those who did not.

The prevalence of heavy drinking was three times higher in Indigenous than non-Indigenous patients. Household surveys demonstrate that while Indigenous Australians are more likely than the rest of the population to be abstainers, those who do drink are more likely to experience alcohol related problems [25]. This greater risk of alcohol-related problems is common to indigenous peoples around the world, and is contributed to by social disadvantage, reduced sense of belonging, reduced control over life, and repeated experiences of loss, such as in early deaths, imprisonment of relatives or separation of children from families [26,27].

Being from a Non-English speaking background makes it less likely that a patient will be a heavy drinker. This may be due to influences of the culture and religion of the country of origin, with both Buddhist and Muslim religions, for example, proscribing intoxication. In addition, families who have been in Australia for shorter periods of time may be less likely to have adopted the pattern of relatively high alcohol consumption that occurs in Australia [28].

The most striking association with heavy drinking in this general practice sample was the higher management rate of psychological problems, depression in particular. After age-sex standardisation, heavy drinkers were 1.6 times more likely to have a psychological problem managed during a visit when alcohol screening took place, although this included the alcohol problem itself (Table 4). Up to 30% of alcohol-dependent patients have depression which often resolves with abstinence and no specific treatment [29]. Counselling about alcohol was provided to the heavy drinking group at a rate of 5.8 per 100 encounters (Table 3). Acute or chronic alcohol problems were managed at a rate of 4.7 per 100 encounters (Table 4). This table includes significant results only. Counselling may have been provided at a higher rate than in typical practice, as the screening questions could have alerted the doctor to an otherwise undetected alcohol problem. However, given this, it is surprising that the management and counselling rates for alcohol related problems were not considerably higher. It is possible that having identified the issue at this encounter, the GPs may follow up at a subsequent encounter. Interestingly, the rate of counselling is almost exactly the same as that reported with general practice patients in another study. In that study the patients were alcohol-dependent and had sought help [30].

Consistent with its association with alcohol, heavy drinkers had a higher management rate of gout. Interestingly, they had lower management rates of ischaemic heart disease and diabetes. Multiple large prospective studies have shown a protective effect of alcohol consumption against ischaemic heart disease, [31] and although heavy drinking is a risk factor for type 2 diabetes, and a number have shown that regular moderate consumption may also be a protective factor with increased insulin sensitivity [32,33]. Alternatively, these results may reflect a tendency for people to avoid heavy drinking after being diagnosed with either of these diseases, whatever their past alcohol consumption behaviour.

The lower management rate of respiratory problems is surprising, given the known association between average daily alcohol consumption and number of cigarettes smoked. However, 80% of the heavy drinkers in this study drank less or not at all on other days and they may not have been regular smokers.

A low rate of detection of risky drinking has been widely reported in both general practice [6,10,34] and hospitals [8,9,35]. This in part reflects the challenges of routine screening, particularly in busy clinical practice [7,36,37]. In Australia, patients are not restricted to one family doctor and may visit a number of different practices. This increases the challenge of quantifying drinking in all new patients. In other cases, lack of routine screening may reflect low GP confidence or skills in detection and intervention for alcohol problems [38-40].

### Limitations of the study

There is a small possibility that a patient was seen twice by the same GP during the BEACH recording period. However the GP may have left the second set of questions blank. Also, as only 40 of the 100 patient encounters are asked the alcohol questions, a patient having a repeat visit may well have been asked a different set of questions at the second visit.

These data cannot claim to reflect the characteristics of the population who attend general practice in Australia. Individuals attend GPs at different rates and the rate is influenced by age, sex and health status as well as socio-economic factors. Therefore, at any single encounter frequent attendees have a greater chance of being included in the study than infrequent attendees. This could lead to an underestimate of the prevalence of heavy drinking in the average GP patient population if problem drinkers attend less often because they neglect their health. Alternately, it could result in an overestimate if problem drinkers have more health problems and attend more often.

The proportion of patients classed as heavy drinkers in this study was lower than the prevalence of 11% detected by CAGE and 24% detected by SMAST in another general practice study [36] and slightly lower than in the general population. Comparison with the National Drug Strategy Household Survey acute risk figures shows an almost identical proportion (7.7%) were drinking at risk of harm in the short term (i.e. consuming >7 drinks for males or >5 drinks for females, on any one day) [1]. The population differs slightly, as the Household Survey also includes people aged 14 to 17 years. The National Drug Strategy Household Survey [1] uses drinks per week as a measure of risk for long-term harm (>28 drinks per week for males or >15 for females is defined as risky). Their figure of 10% is comparable with our rate of 7.3%. Our prevalence of heavy drinking is considerably lower than that from the New South Wales Adult Health Survey, [41] which estimates that 34% of the people who drink alcohol report some risk drinking behaviour. However, that survey excludes non-drinkers.

The screening question used in this analysis would not have detected men or women drinking over the Australian recommended limit but below 6 drinks per occasion (i.e. women drinking 3–5 drinks daily, or men 5 drinks daily). It is also possible that alcohol problems were under-detected, due to deliberate patient under-reporting or embarrassment at reporting their true rate of consumption, or because drinking was inadvertently not reported in standard drinks (a can of beer, for example is 1.5 Australian standard drinks), although a standard drinks chart was provided for reference.

In other settings, asking this one question (How often do you drink six or more drinks on one occasion?) has been previously demonstrated to be a highly specific screen (94–99%) in detecting problem drinkers identified by a validated 10-item questionnaire. The sensitivity was somewhat lower, ranging from 79–89% [19,20] but still considerably higher than the 30%–45% sensitivity that has been obtained by doctors in routine clinical practice [6,7,18].

### Implications

The prevalence of heavy drinking found in this study in patients attending general practice provides GPs with about 7.3 million opportunities per year in Australia, during normal consultations with their patients, to identify these issues and provide advice or counselling. This study illustrates the wide range of presentations of heavy drinkers, the need for a high level of alertness to the possibility of alcohol problems, and for a routine brief alcohol history.

Given time pressures faced by GPs and past poor detection rates, there is ongoing need for efficient methods of screening for alcohol problems. The single item screening question used in this study has previously been shown to have high specificity, but imperfect sensitivity. The three item AUDIT-C questionnaire (sensitivity 92%, specificity 74% for males; 91% and 68% for females) [42] provides an acceptable and still brief alternative, as do the variety of 4–6 item questionnaires developed for rapid detection of alcohol problems [43,44]. In some countries, practice nurses take on the role of screening for health risk factors [45,46]. In addition, time saving techniques such as waiting- room screening, sometimes using handheld computers, are being investigated.

## Conclusion

Alcohol problems are common and have a major impact on the workload of the family doctor. While traditional teaching in medical schools focused on alcohol-related end organ damage, patients with alcohol problems are more likely to have depression and other psychological problems, physical injuries and gout managed by their GP. However, the wide range of morbidity managed in heavy drinkers means that relying on clinical impression alone to detect this group will not suffice. Routine alcohol history-taking or other screening is required. Given the pressures of general practice, finding efficient methods of screening for alcohol problems remains a priority.

## Competing interests

The author(s) declare that they have no competing interests.

The authors: HB is the Director of the AIHW Australian General Practice Statistics & Classification Centre and had the idea for the paper; LV, Senior Analyst, analysed the data; and EP and KM wrote the first draft of the paper. All authors contributed substantially to the writing and the revisions of the article.

## Pre-publication history

The pre-publication history for this paper can be accessed here:


